# Popliteal Artery Entrapment Syndrome as a Differential Diagnosis for Knee Pain: A Case Report

**DOI:** 10.1055/s-0044-1779307

**Published:** 2024-04-24

**Authors:** Antuny Rodrigues Rosa, Hamilton Santos Cé, Ana Clara Monteiro Marchini, Anna Katarina Menegon Lopetegui, Anna Luiza Lunardelli Padilha, Gustavo Rosa Bianchini

**Affiliations:** 1 Hospital Nossa Senhora dos Prazeres, Lages, SC, Brasil; 2 Hospital Tereza Ramos, Lages, SC, Brasil; 3 Universidade do Planalto Catarinense, Lages, SC, Brasil

**Keywords:** knee, pain, popliteal artery entrapment syndrome

## Abstract

Popliteal artery entrapment syndrome has congenital and functional causes. It mostly affects young people. There are six types of popliteal artery entrapment syndrome. Here, we report a case of type III popliteal artery entrapment syndrome, with an anatomical variation of the gastrocnemius muscle, with an accessory band laterally attached in the femoral condyle compressing the popliteal artery. The patient had characteristic symptoms, with intermittent claudication, paresthesia, absence of blood flow in the posterior tibial and dorsalis pedis arteries during dorsiflexion and plantar flexion of the right foot, and pain in the posterior knee at the physical examination. Imaging supplemented the diagnosis, and the method of choice was magnetic resonance imaging to identify vascular alterations in structures adjacent to the blood vessels in the popliteal fossa. The treatment was surgical for symptom relief and complication prevention. The condition improved with the resection of the accessory band of the gastrocnemius muscle. It is worth noting that the existing literature on the subject is scarce, but the approach adopted here is consistent with other publications. The present report is critical for understanding the popliteal artery entrapment syndrome as a differential diagnosis for knee pain.

## Introduction

Popliteal artery entrapment syndrome (PAES) results from obliteration of the popliteal arterial flow by adjacent structures, causing pain and low blood perfusion in the affected limb. 1 Its etiology is both anatomical (congenital) and functional. In congenital PAES, there is a vascular malformation during the embryonic period or the abnormal development of structures surrounding the artery, reducing the blood flow in its lumen. In contrast, functional PAES results from the hypertrophy of the musculature around the artery and its consequent compression. 2 The present paper addresses PAES diagnosis and treatment based on a case of a patient who agreed with the report by signing an informed consent form (ICF).

## Clinical Case

A 20-year-old Caucasian woman came to an orthopedic specialty outpatient clinic complaining of pain in her right knee for one year, which had worsened for one month. At that time, the most likely diagnostic hypothesis was patellofemoral pain syndrome. For diagnosis clarification, a magnetic resonance imaging (MRI) scan of the right knee was requested; it revealed an anatomical variation in the popliteal fossa of the accessory band at the attachment site of the lateral gastrocnemius, which caused entrapment of the popliteal artery and the symptoms presented by the patient. This piece of information led to the suspicion of PAES, and the patient was referred to a vascular surgeon for clinical tests and adequate treatment planning.

During consultation with the vascular surgeon, specifically during the physical examination, the patient presented intermittent claudication in the right lower limb. Palpation of posterior tibial and dorsalis pedis arterial pulses on the right and left sides revealed symmetrical, full, and rhythmic pulses at rest. However, dorsiflexion and plantar flexion of the right foot led to the disappearance of the posterior tibial and dorsalis pedis pulses at palpation and digital pallor, which did not occur in the contralateral limb. A physical examination of the knee showed no other particularities.

**Fig. 1:**
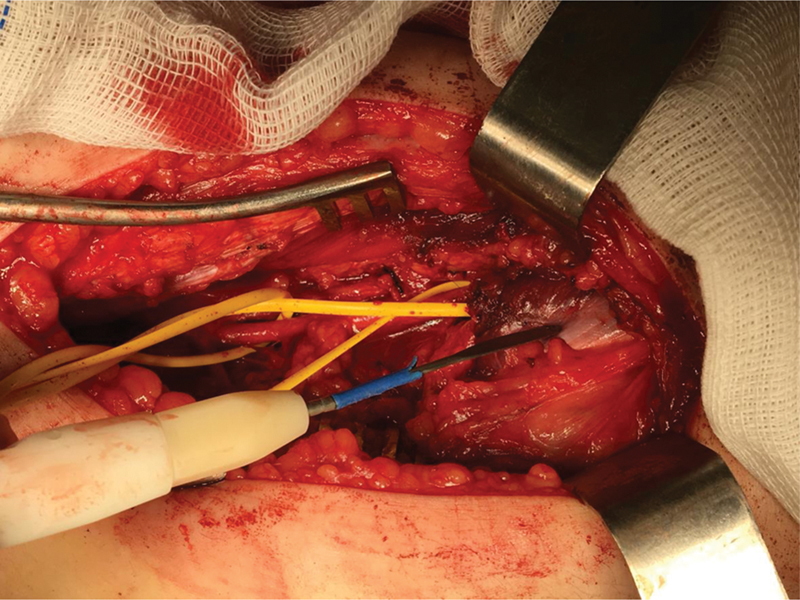
Posterior view of the knee. Popliteal fossa approach through an S-shaped incision. Popliteal artery entrapped by an accessory band of the lateral gastrocnemius.

**Fig. 2:**
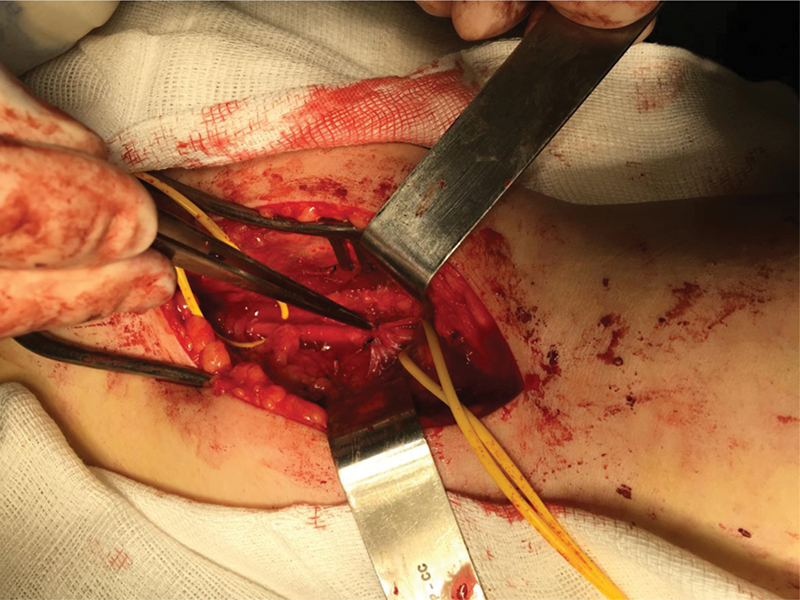
Posterior view of the knee. Extrication of the popliteal artery with resection of the accessory band of the lateral gastrocnemius.

**Fig. 3:**
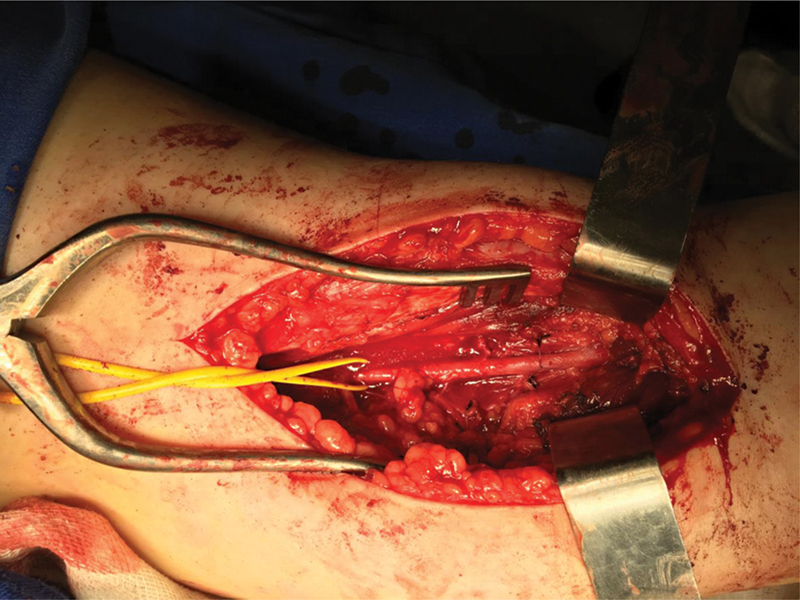
Posterior view of the knee. Resolution of the popliteal artery entrapment by resecting the accessory band of the lateral gastrocnemius.

After the diagnosis, we decided on surgical treatment. With the patient in the prone position, we made an S-shaped incision in the popliteal fossa for a posterior approach to identify the popliteal artery and vein and the sural nerve ( Fig. 1 ). Then, we resected the accessory muscle band of the lateral gastrocnemius ( Fig. 2 ) and released the entrapment of the corresponding popliteal artery ( Fig. 3 ).

The surgical treatment was uneventful. During the postoperative follow-up, a physical examination revealed no changes in the right dorsalis pedis and posterior tibial pulses during plantar dorsiflexion. Furthermore, 30 days after surgery, a Doppler ultrasonography of the popliteal vessels with plantar flexion forced against resistance showed satisfactory recovery and postsurgical follow-up. Therefore, these tests were repeated 6 and 12 months after surgery, and the prognosis was good, since treatment was successful. In summary, the patient had a satisfactory postoperative period, with symptom remission and return to daily activities, including sports, resulting in improved quality of life.

## Discussion

Popliteal artery entrapment syndrome is the leading cause of intermittent claudication in young patients without atherosclerotic disease etiologies, 2 3 and one of the differential diagnoses of lower limb pain, along with tibial stress syndrome, stress fractures, arteritis, myopathies, popliteal artery cyst, and tendinopathies. 1 4

Popliteal artery entrapment results in significant symptoms in the lower limbs, including pain, paresthesia, and physical exertion-triggered pallor. 2 4 Lamônica et al. 5 demonstrated that semiological maneuvers of foot dorsiflexion or flexion caused reduced or absent posterior tibial and dorsalis pedis pulses due to compression of the popliteal artery from the contraction of the adjacent muscles, as shown in the physical examination of our patient.

Among the supplementary diagnostic tests available, MRI is more effective for the arteriographic study and the analysis of structures adjacent to vessels in the popliteal fossa compared with other imaging methods, such as computed tomography (CT). Magnetic resonance imaging revealed an anatomical variation in our patient, which enabled us to determine the diagnosis the diagnosis of PAES, classify it, and select the appropriate treatment. There are six types of PAES, 2 5 namely:

- Type I: Popliteal artery with medial deviation but normal attachment of the gastrocnemius muscle in the internal condyle of the femur.
- Type II: Popliteal artery with regular course, passing anteriorly to the internal tendon of the gastrocnemius muscle, which is attached more laterally into the internal condyle of the femur.
- Type III: The gastrocnemius muscle has an anatomical variation, that is, an additional tendon attached laterally, compressing the artery.
- Type IV: Arterial compression by the popliteus muscle, with no anatomical variations of the gastrocnemius muscle.
- Type V: Concomitant compression of the popliteal artery and vein.
- Type VI: Functional arterial compression by muscle hypertrophy with regular constitution.

The case herein reported is classified as type III since the MRI showed an anatomical variation, that is, an accessory band at the origin of the lateral gastrocnemius, which compresses the popliteal artery against the femoral condyle during muscle contraction. 4

It is worth noting that surgery is the treatment of choice for popliteal artery extrication even in asymptomatic patients 2 because of PAES complications, including arterial thrombosis from repeated vascular damage, thromboembolism, or vascular aneurysm. 1

Furthermore, the approach to the popliteal fossa occurs through an S-shaped incision to fold the popliteal face after surgery. 4 In type-III PAES, after identifying the accessory band, it is necessary to perform its resection to extricate the popliteal artery. 3

The present case report shows the significant role of surgical treatment in decompressing the popliteal artery to alleviate symptoms and prevent potential complications. Considering the scarce literature on the subject, the present case report can serve as a reference to guide professionals in the diagnosis and treatment of intermittent claudication and knee pain in young patients, since PAES is one of the differential diagnoses.
