# Supplementary material for: Popliteal Artery Entrapment Syndrome as a Differential Diagnosis for Knee Pain: A Case Report
Source: Rev Bras Ortop (Sao Paulo). 2024 Apr 24;59(Suppl 2):e159–62. doi: 10.1055/s-0044-1779307 (PMC11679624; doi:10.1055/s-0044-1779307)
Supplement: Related texts

# Research article: Síndrome de aprisionamento da artéria poplítea como diagnóstico diferencial de dor no joelho: Um relato de caso

**Keywords:** dor, joelho, síndrome de aprisionamento da artéria poplítea

## Abstract

A síndrome do aprisionamento da artéria poplítea possui causas congênitas e funcionais, acometendo, em grande parte, a população mais jovem. Existem seis tipos de síndrome, sendo o caso relatado classificado como tipo III, isto é, o músculo gastrocnêmio apresenta variação anatômica, com banda acessória que se insere lateralmente no côndilo femoral, comprimindo a artéria poplítea. A paciente apresentou sintomatologia característica, com claudicação intermitente, parestesia e ausência de fluxo sanguíneo nas artérias tibiais posteriores e pediosas quando submetida à dorsiflexão e flexão plantar do pé direito, além de dor em face posterior do joelho durante a realização do exame físico. O diagnóstico foi complementado por exames de imagem, sendo o método de escolha a ressonância magnética, capaz de identificar alterações vasculares nas estruturas adjacentes à vasculatura na fossa poplítea. O tratamento é cirúrgico e visa tanto o alívio dos sintomas quanto a prevenção de complicações, pois, a ressecção da banda acessória do músculo gastrocnêmio permitiu boa evolução do quadro da paciente. Há que se pontuar que a literatura existente sobre o tema é escassa, mas a conduta adotada no caso em questão converge com as demais publicações encontradas. O relato apresentado é importante para a compreensão da síndrome como diagnóstico diferencial da dor no joelho.

## Introdução

A síndrome de aprisionamento da artéria poplítea (SAAP) é caracterizada pela obliteração do fluxo arterial poplíteo por estruturas adjacentes a esse vaso sanguíneo, ocasionando dor e baixa perfusão sanguínea no membro acometido. 1 Sua etiologia é conhecida tanto por causas anatômicas (congênitas) quanto por causas funcionais. Dessa forma, quando a causa é congênita, há má-formação vascular durante o período embrionário ou ainda desenvolvimento anormal de estruturas que circundam a artéria, reduzindo o fluxo sanguíneo em sua luz. Por sua vez, a causa funcional é atribuída à hipertrofia da musculatura ao redor da artéria, comprimindo-a. 2 Primordialmente, no presente artigo, serão abordados o diagnóstico e o tratamento dessa síndrome a partir de um relato de caso consentido pela paciente, por meio da assinatura do Termo de Consentimento Livre e Esclarecido (TCLE).

## Caso Clínico

Uma mulher branca de 20 anos de idade procurou um ambulatório de especialidades ortopédicas com queixa de dor no joelho direito há um ano, com agravamento há um mês. Nesse momento, a hipótese diagnóstica mais provável era a síndrome da dor patelofemoral. Para elucidar o diagnóstico, optou-se pela realização de uma ressonância magnética (RM) do joelho direito, que apontou variação anatômica na fossa poplítea de banda acessória na origem do gastrocnêmio lateral, levando ao aprisionamento da artéria poplítea e à sintomatologia apresentada pela paciente. Com base nessas informações, a hipótese diagnóstica de SAAP foi levantada e a paciente foi encaminhada para um cirurgião vascular para testes clínicos e planejamento do tratamento adequado.

Durante a consulta com o cirurgião vascular, mais especificamente no exame físico, a paciente apresentou claudicação intermitente no membro inferior direito durante a marcha. Ao realizar a palpação dos pulsos arteriais tibiais posteriores e pediosos, tanto do lado direito quanto do esquerdo, observou-se que os pulsos eram simétricos, cheios e rítmicos quando os membros estavam em repouso. No entanto, ao realizar a dorsiflexão e flexão plantar do pé direito, houve desaparecimento do pulso tibial posterior e pedioso à palpação, acompanhado de palidez nos pododáctilos, o que não foi observado no membro contralateral. O exame físico do joelho não apresentou outras particularidades.

Após o diagnóstico, optou-se pelo tratamento cirúrgico. Com a paciente em decúbito ventral, realizou-se uma incisão em forma de S na fossa poplítea para obter acesso posterior, identificando a artéria e a veia poplítea, bem como o nervo sural ( Fig. 1 ). Em seguida, foi feita a ressecção da banda muscular acessória do gastrocnêmio lateral ( Fig. 2 ) e o desencarceramento da artéria poplítea correspondente ( Fig. 3 ).

**Fig. 1:** Vista posterior do joelho. Acesso da fossa poplítea por incisão em S. Artéria poplítea encarcerada por banda acessória do gastrocnêmio lateral.

**Fig. 2:** Vista posterior do joelho. Desencarceramento da artéria poplítea com ressecção da banda acessória do gastrocnêmio lateral.

**Fig. 3:** Vista posterior do joelho. Artéria poplítea desencarcerada após ressecção de banda acessória do gastrocnêmio lateral.

Após a realização do tratamento cirúrgico, que transcorreu sem intercorrências, iniciou-se o acompanhamento pós-operatório da paciente. E, no exame físico, observou-se que os pulsos pediosos e tibial posterior direitos estavam sem alterações à dorsiflexão plantar. Outrossim, após 30 dias da cirurgia, foi realizada ultrassonografia com Doppler dos vasos poplíteos com flexão plantar forçada contra resistência, constatando-se boa recuperação da paciente e bom segmento pós-cirúrgico. Por conseguinte, os exames foram repetidos após 6 e 12 meses da intervenção cirúrgica, e o prognóstico foi considerado bom, evidenciando sucesso no tratamento. Em resumo, a paciente teve um bom segmento pós-operatório, com remissão dos sintomas e retorno às atividades diárias, inclusive hábitos esportivos, resultando em melhora na qualidade de vida.

## Discussão

Considerada a principal causa de claudicação intermitente em pacientes jovens sem outras etiologias de doenças ateroscleróticas, 2 3 a SAAP é um dos diagnósticos diferenciais da dor em membro inferior, juntamente com a síndrome do estresse tibial, fraturas por estresse, arterite, miopatias, doença cística da artéria poplítea e tendinopatias. 1 4

Por conseguinte, o encarceramento da artéria poplítea provoca sintomatologia importante nos membros inferiores, como dor, parestesia e palidez desencadeados após esforço físico. 2 4 Lamônica et al. 5 demonstram em seu estudo que, durante a realização de manobras semiológicas de dorsiflexão ou flexão plantar do pé, ocorre a redução ou ausência de pulso tibial posterior e pedioso por compressão da artéria poplítea a partir da contração dos músculos adjacentes a esta, o que ficou demonstrado no exame físico da paciente do presente caso.

Dentre os métodos diagnósticos complementares disponíveis, a RM tem se mostrado mais eficaz tanto no estudo arteriográfico quanto na análise das estruturas adjacentes aos vasos da fossa poplítea, em comparação com outros métodos de imagem, como a tomografia computadorizada (TC), por exemplo ^[1]^ . Por meio deste exame, foi possível determinar a variação anatômica que acometia a paciente em questão, permitindo o diagnóstico e a classificação da SAAP, bem como a definição do tratamento adequado. A SAAP é classificada em seis tipos, 2 5 a saber:

- Tipo I: Artéria poplítea com desvio medial, mas com inserção normal do músculo gastrocnêmio em côndilo interno do fêmur.
- Tipo II: Artéria poplítea com trajeto normal, passando anteriormente ao tendão interno do músculo gastrocnêmio, que se insere mais lateralmente ao côndilo interno do fêmur.
- Tipo III: Músculo gastrocnêmio apresenta variação anatômica, com tendão adicional que se insere lateralmente, comprimindo a artéria.
- Tipo IV: Compressão arterial pelo músculo poplíteo, sem alterações anatômicas do músculo gastrocnêmio.
- Tipo V: Compressão concomitante da artéria e veia poplítea.
- Tipo VI: compressão funcional das artérias pela hipertrofia muscular com constituição normal.

O caso relatado é classificado como tipo III, já que a RM demonstrou variação anatômica de banda acessória na origem do gastrocnêmio lateral, a qual comprime a artéria poplítea contra o côndilo femoral quando o músculo se contrai. 4

Há que se pontuar que a terapêutica cirúrgica é o tratamento de escolha para promover o desencarceramento da artéria poplítea, mesmo em pacientes assintomáticos, 2 dada as complicações da síndrome, como trombose arterial a partir do dano vascular repetido, tromboembolismo ou aneurisma vascular. 1

Ademais, o acesso à fossa poplítea é feito com uma incisão em “S” para permitir o pregueamento da face poplítea após a cirurgia. 4 Uma vez identificada a banda acessória, na SAAP tipo III, é necessário realizar a sua ressecção para desencarcerar a artéria poplítea. 3

A partir do presente caso, fica evidente a importância do tratamento cirúrgico para descomprimir a artéria poplítea, tanto para aliviar os sintomas quanto para prevenir possíveis complicações. Considerando a escassa literatura disponível sobre o assunto, o presente relato de caso pode servir como referência para orientar os profissionais no diagnóstico e tratamento da claudicação intermitente e dor no joelho em pacientes jovens, tendo em vista que a SAAP é um dos diagnósticos diferenciais a serem considerados.
